# A Congenital Spinal Epidermoid Cyst

**DOI:** 10.5334/jbr-btr.848

**Published:** 2015-09-15

**Authors:** L. Morbée, Ph. Lagae, B. Smet, N. Baelde, J. De Mey

**Affiliations:** 1 Department of Radiology, University Hospital Brussels, Belgium; 2 Department of Radiology, AZ Jan Palfijn, Ghent, Belgium; 3 Department of Radiology, University Hospital Ghent, Belgium

A 73-year-old female with unresolved digestive complaints was referred to our department for CT imaging of the abdomen. Unexpectedly, an oval-shaped, well delineated, intramedullary located mass was found in the spinal canal at the level of the vertebra D11 and D12. On CT imaging the mass is hyperdense to CSF, shows intramural calcification and minimal to no contrast enhancement (Fig. 1A). Proximal to the spinal mass there is a longitudinal split in the spinal cord, diastematomyelia, associated with a congenital block vertebra and hemivertebra at the level of D9–D10 (Fig. 1B). There is also an unfused spinous process of D9 and D10, an anatomical variant which is part of the spectrum of spina bifida occulta (Fig. 1C). The patient has no symptoms of low back pain and no abnormalities in the neurological clinical examination.

**Figure 1:**
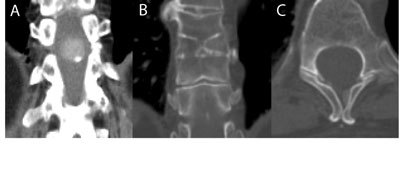


MR imaging is required for further differentiation of the mass and shows a well-defined lesion without perilesional edema, with hyperintense signal on T1 weighted images (Fig. 2A), inhomogeneous, isointense to hypointense signal on T2 weighted images (Fig. 2B) and no significant enhancement after contrast administration.

**Figure 2:**
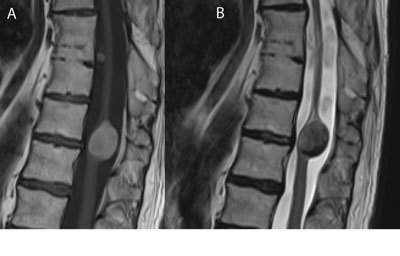


For further differentiation diffusion weighted imaging (Fig. 3A) was performed and revealed diffusion restriction with corresponding low intensity on ADC map (Fig. 3B) in the entire mass, except in the region of calcification. These findings, in association with the spinal malformations (spina bifida, diastematomyelia, block vertebra and hemivertebra) are highly specific for a congenital spinal epidermoid cyst. The diastematomyelia is best shown on the coronal T2 weighted image in Fig. 4.

**Figure 3:**
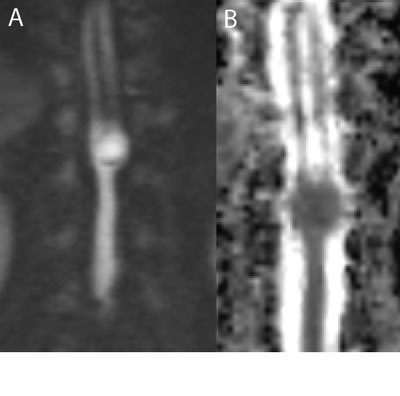


**Figure 4:**
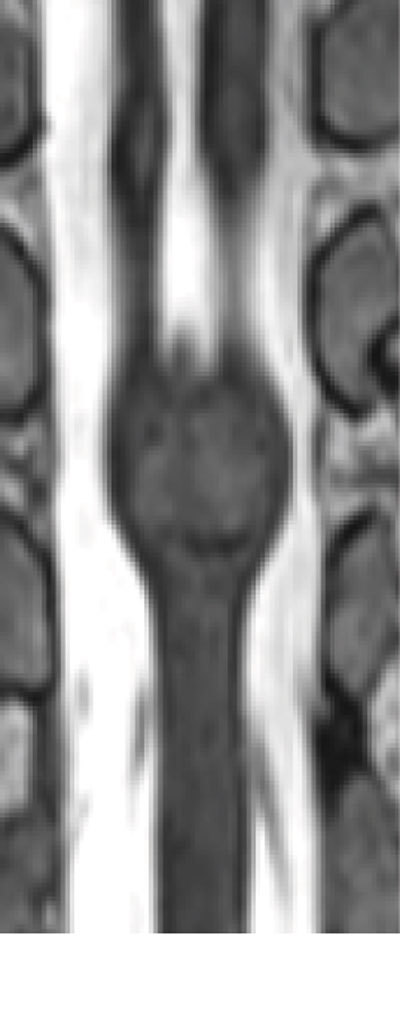


## Comment

A spinal epidermoid cyst is a benign nonneoplastic spinal mass embryologically derived from epidermal (skin) elements. Unlike dermoid cysts, they do not contain skin appendages (hair follicles, sweat glands, sebaceous glands). Histologically, they consist of stratified squamous epithelium supported by an outer layer of collagenous tissue. Progressive desquamation and breakdown of keratin from the epithelial lining into the interior of the cyst produce the characteristic content.

Generally epidermoid cysts are hypodense and hypointense masses with attenuation similar to CSF. Signal intensity of epidermoid cysts on T1 and T2 weighted images may be homogeneous or heterogeneous according to the variable water, lipid and protein composition of the cyst. The high signal on T1 weighted image in this case is reflecting high protein content or cellular debris.

Spinal epidermoid cysts are uncommon. They comprise between 0.5% and 1% of all spinal tumors, but account for up to 10% of intraspinal tumors in children. 40% is intramedullary located. Although present since birth, congenital epidermoid tumors are often not discovered until the second to fourth decade of life (in this case 8^th^ decade), since they are mostly asymptomatic. If symptomatic, motor disturbances, pain, sensory disturbances, and bowel or bladder dysfunction may be present. Epidermoid cysts are commonly associated with spinal malformations such as spina bifida and hemivertebrae. Congenital spinal epidermoids result from anomalous implantation of ectodermal cells during closure of the neural tube between the third and fifth week of embryonic life.

The top differential diagnosis of an epidermoid cyst inludes a spinal arachnoid cyst, dermoid cyst and neurenteric cyst. Diffusion restriction is the best way of distinguishing an epidermoid cyst from these cysts. Dermoid cysts are less likely to show diffusion restriction and arachnoid cysts never do so. Vertebral anomalies are uncommon in these two. Spinal neurenteric cysts can be associated with vertebral anomalies. They are intradural cysts usually located ventral to the spinal cord, affecting the thoracic and cervical regions.

Spinal epidermoid cysts are slow-growing and surgery is the treatment of choice. Complete excision is usually possible and is curative. The patient in this case chose not to be operated, given her age, the lack of symptoms and anticoagulant drug therapy.

## Competing Interests

The authors declare that they have no competing interests.

## References

[1] Agarwal A, Bhake A, Kakani A (2011). Cervical intramedullary epidermoid cyst with liquid contents. Asian Spine Journal.

